# Research on Image Reconstruction of Compressed Sensing Based on a Multi-Feature Residual Network

**DOI:** 10.3390/s20154202

**Published:** 2020-07-28

**Authors:** Ruili Nan, Guiling Sun, Zhihong Wang, Xiangnan Ren

**Affiliations:** College of Electronic Information and Optical Engineering, Nankai University, Tianjin 300350, China; 1611206@mail.nankai.edu.cn (R.N.); wanghao801226@nankai.edu.cn (Z.W.); 1611101@mail.nankai.edu.cn (X.R.)

**Keywords:** image reconstruction, compressed sensing, multi-feature, residual block, deep learning

## Abstract

In order to solve the problem of how to quickly and accurately obtain crop images during crop growth monitoring, this paper proposes a deep compressed sensing image reconstruction method based on a multi-feature residual network. In this method, the initial reconstructed image obtained by linear mapping is input to a multi-feature residual reconstruction network, and multi-scale convolution is used to autonomously learn different features of the crop image to realize deep reconstruction of the image, and complete the inverse solution of compressed sensing. Compared with traditional image reconstruction methods, the deep learning-based method relaxes the assumptions about the sparsity of the original crop image and converts multiple iterations into deep neural network calculations to obtain higher accuracy. The experimental results show that the compressed sensing image reconstruction method based on the multi-feature residual network proposed in this paper can improve the quality of crop image reconstruction.

## 1. Introduction

Crops will inevitably be affected by diseases, insects, soil conditions, climate and other factors during the growth process. Realizing the monitoring of crop growth is not only an important means for constructing agricultural information, but it can also improve bad factors in time. The image is a direct means to perceive and analyze the growth situation of crops. In addition to analyzing the growth changes of leaves and rhizomes of crops, it can also determine the maturity of crops. Accurate image acquisition is the prerequisite for image processing and analysis.

The image acquisition task needs to be completed by the wireless sensor network node, whose main feature is to rely on battery power supply and limited energy. In the transmission and storage of large data images, it will face problems such as resource constraints, which will easily lead to a short life cycle of the node. Therefore, how to ensure the quality and quantity of image information acquisition in an environment with limited energy and resources has become a problem that needs to be solved urgently. In short, in order to make the data collection of sensor nodes achieve the effects of greatly reducing the amount of data and network energy consumption, effective information compression has become the focus of research.

**Figure 1:**
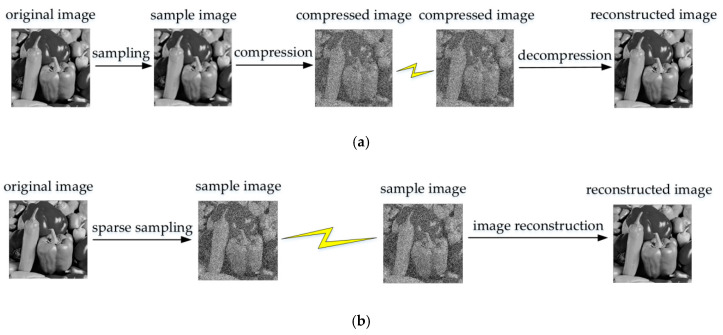
The comparison between the two modes: (**a**) traditional image acquisition mode; (**b**) image acquisition mode based on sparse sampling.

In 2006, Donoho et al. [1,2] proposed the theory of compressed sensing (CS), CS sampling breaks through the limitations of the Nyquist sampling theorem. Random sampling is used to obtain discrete signal samples at a low sampling rate. The traditional signal sampling and compression processes are combined into one, and then the signal is nonlinearly reconstructed by discrete samples; it provides a solution for the data transmission effect of low energy consumption, low storage, low broadband, and high data volume. Compared with traditional sampling and compression techniques, the image acquisition method based on compressed sensing has the advantages of simple coding and good compression performance. The comparison between the two modes is shown in Figure 1.

The reconstruction problem is one of the research focuses in the field of CS. Since CS image reconstruction is the prerequisite for accurate judgment and analysis, our article mainly explores its compressed sensing image reconstruction in depth. Traditional compressed sensing systems usually use convex optimization or iterative algorithms for signal recovery under the premise of sparse signal. For crop images whose background environment are more complicated, however, they do not accurately meet the sparsity in the transform domain, and it takes a long time to solve the optimization problem based on iterative solutions, making it difficult to process image information quickly.

In recent years, deep learning has developed rapidly in various fields and has been widely used. It has achieved significant performance improvements in many traditional signal representation and recognition tasks, showing its ability to handle complex recognition tasks. Introducing deep learning technology into CS image reconstruction provides a better solution to the problems of traditional CS reconstruction with high time-consuming and low image reconstruction accuracy when the measurement rate is low. Deep learning uses a large amount of data and data labels, namely compressed images and original images, to learn the structural characteristics of crop image information adaptively; meanwhile, it converts the traditional measurement and reconstruction process into an end-to-end mapping network, reducing network complexity and measurement parameters. With the rapid development of the Internet and the advent of the era of big data, highly parallel graphics processing unit (GPU) and massive amounts of data ensure network computing time and image quality.

DeepCodec [3] learns a transformation from the original signals to a near-optimal number of undersampled measurements and the inverse transformation from measurements to signals. CSNet [4] proposes a deep network to recover the image, which imitates traditional compressed sensing reconstruction processes. DeepInverse [5] uses a deep convolutional neural network to learn signal structure to solve the problem of incomplete sparse real data and slow reconstruction algorithm convergence under a fixed change basis. DR2-Net [6] uses the residual module to solve the problem of deep network degradation and achieve image reconstruction at low measurement rate. Bora et al. [7,8,9,10] used generative models to construct deep learning frameworks to solve compressed sensing reconstruction problems. CSRNet [11] uses the residual network module based on a convolutional neural network to enhance the initial image of deep reconstruction. Zhang et al. [12] proposed a structured deep network ISTA-Net, which expands the traditional compressed sensing iterative shrinkage threshold algorithm into a deep learning network. Yochai Zur et al. [13] proposed an end-to-end deep learning algorithm that uses structural similarity (SSIM) as a training loss function to jointly optimize the perception matrix and nonlinear reconstruction operator. Zhou et al. [14] proposed a multi-channel deep neural network structure that improves the quality of reconstructed images by iterative block approximation and denoising based on the entire image. MSRNet [15] is an end-to-end multi-scale residual convolutional neural network; it is used to simulate the real image compression perception and inverse reconstruction process.

Based on the existing algorithms, we proposes a compressed sensing image reconstruction method based on a multi-feature residual network that uses multi-scale convolution to obtain different feature information of crop images. The residual block can solve the problem of deep neural network degradation well, avoiding the gradient dispersion problem caused by the deepening of the network. The multi-feature extraction of the image can capture more different image details, so that the image information is more completely retained, and the image reconstruction ability of the network is improved.

In summary, this paper studies the problem of image acquisition in the process of crop growth monitoring, and researches the inverse solution of compressed sensing to achieve higher quality reconstruction of crop images. On the one hand, it provides a reliable basis for the next image analysis and other processing. During the monitoring of the growth process of crops, problems such as withering of branches and leaves, falling of fruits, moth eaten etc. are discovered as soon as possible; thereby permitting timely harvesting of fruits when they are ripe. On the other hand, in the traceability of agricultural products, it provides real and effective image display to realize the visual presentation of its growth process. With a small amount of data, low energy consumption and high speed of transmission, CS sampling extends the life of sensor nodes, allowing them to monitor crop growth for a longer time; the CS reconstruction algorithm based on deep learning uses massive data to learn image features autonomously, which can speed up the reconstruction process. The experimental results show that compressed sensing image reconstruction based on multi-feature residual network (MRNet) proposed in our paper can effectively improve the accuracy of crop image reconstruction, indicating that the multi-feature extraction of images has a good effect on improving the quality of reconstructed images. It can promote the application of wireless sensor networks (WSNs) in crop growth monitoring, accelerate the development of intelligent Internet of Things and improve the construction of agricultural informatization.

## 2. Compressed Sensing and Deep Learning

### 2.1. Compressed Sensing

The measured value containing the original image information is obtained by the measurement matrix, and then the original image can be obtained by the reconstruction algorithm. We suppose that *x* is an *N* × *1* sparse signal that has only *k* nonzero values, and then we can recover *x* from a set of under sampled linear measurements *y* = Φ*x* (*y* ∈ *R^M^*), the Φ denotes the random measurement matrix (Φ ∈ *R^M^^× N^*, M≪N).

**Figure 2:**
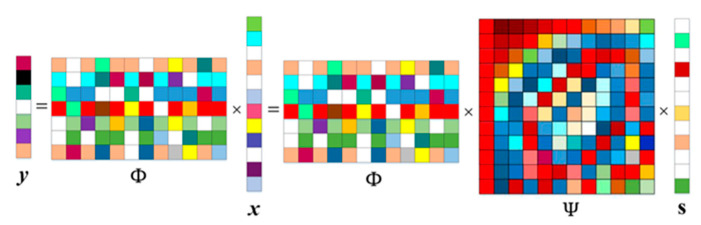
The schematic of compressed sensing.

However, since the general natural signal *x* does not satisfy sparseness, it needs to be sparsely expressed on the sparse basis; let *x* = Ψ*s*, Ψ∈ *R^N^^×N^* is the sparse basis matrix, and *s* is the sparse coefficient. The mathematical expression of compressed sensing is shown in Equation (1), and the schematic diagram is shown in Figure 2.

*y* = Φ*x =* Φ Ψ*s*. (1)

Among them, the sparse signal measurement matrix Φ must meet the restricted isometry property (RIP) [1,16] in pursuit of higher quality, which can ensure that the measurement matrix does not map two different sparse signal s into the same set; in other words, it ensures a one-to-one mapping relationship between the original space and the sparse space, thereby ensuring that the signal can be accurately reconstructed from the measured values. Although it is difficult to construct the measurement matrix simply by RIP conditions, the incoherence between the measurement matrix Φ and the sparse basis matrix Ψ can make the measurement matrix Φ with high probability to satisfy the RIP.

The essence of the compressed sensing image reconstruction problem is to solve an non-deterministic polynomial (NP-hard) problem, which can be achieved by:

(2) $$s=\min_{s}\|s{\|}_{0}s.t.\|y-\Phi \Psi s\|\leq \varepsilon$$

where *ε* is a constant approaching 0. Since the solution of Equation (2) is a NP-hard problem, it cannot be solved accurately; what we can do is make the estimated value approach the true value indefinitely. The traditional compressed sensing reconstruction algorithm mainly includes the following three categories:

1. Convex optimization algorithm: uses $\ell_{1}$ norm approximation instead of $\ell_{0}$ norm, and convert the nonconvex problem to convex problem to solve the infinite approximation of signal, such as the gradient projection method (GPSR).
2. Greedy tracking algorithm: selects the local optimal solution through iterations to approximate the original signal, for example, by using the subspace tracking algorithm (SP) [17,18].
3. Nonconvex optimization algorithm: such as the Bayesian compressed sensing algorithm (BCS), based on statistics, the prior probability density distribution function of the signal is obtained from the prior knowledge. Then, the maximum posterior probability is used to estimate the error range of the reconstructed value and finally reconstruct the original signal.

### 2.2. Compressed Sensing Reconstruction Based on Deep Learning

The reconstruction method based on deep learning uses a large amount of data and data labels, namely compressed images and original images, by continuously adjusting network weights adaptively and offsets to learn the structural characteristics of crop image information, and how to use the data structure to accelerate the reconstruction process. The reconstruction steps are as follows:

**Step 1:** Data preparation.

Load the dataset and preprocess the image, and then construct a measurement matrix for compressed sensing sampling to obtain a compressed crop image. Correspond this to the initial crop image and the compressed image to obtain data and data labels.

**Step 2:** Input data and labels to reconstruct the network.

Input the data and labels to the reconstruct the network and update the network parameters, and dynamically adjust the learning rate through the optimizer, thereby minimizing the loss function.

**Step 3**: Test.

When the training end condition is reached, save the network parameters, load the test image into the trained reconstruction network, calculate the peak signal to noise ratio between the reconstruction and the original image to evaluate the image quality, and then end the training.

## 3. Image Reconstruction Based on a Multi-Feature Residual Network

To address the problem of how to quickly and accurately obtain crop images during crop growth monitoring, this paper proposes a compressed sensing image reconstruction algorithm based on a multi-feature residual network. We use this fully connected to form a linear mapping network, and use multi-scale convolution to achieve multi-feature extraction of images, so that the reconstruction network can make full use of the structural features of crop image information, and train network parameters, aiming to improve the quality of reconstructed images by multi-feature fusion.

### 3.1. Data Prepare

**Figure 3:**
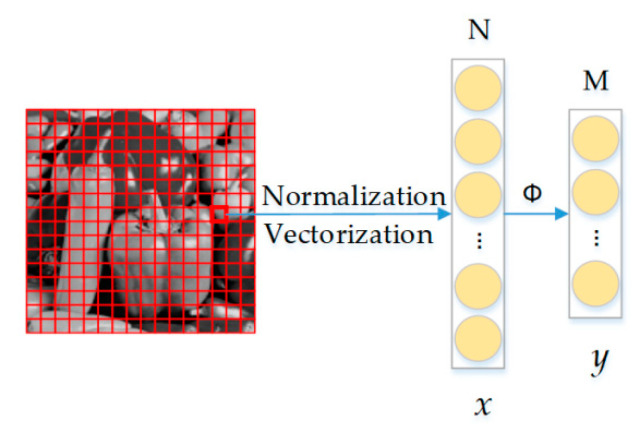
The process diagram of data preparation.

Before the data are transferred to the reconstruction network, data preprocessing is needed. The implementation process is shown in Figure 3.

First, for the convenience of subsequent data processing and to ensure the convergence speed of the model during operation, the original image is normalized. The mathematical model is shown in Equation (3):

(3) $$x^{\prime }=\frac{x-x_{\min}}{x_{\max}-x_{\min}},$$

where *x*, *x*′, *x*_max_, and *x*_min_ represent the original pixel value, the normalized pixel value, the maximum pixel value, and minimum pixel value, respectively.

Secondly, divide all training set images into n × n subimage blocks and vectorize them to obtain the signal *x* of N = n^2^ dimension, which can effectively avoid the problem of excessive data in the subsequent initial reconstruction process, reduce the number of network parameters, and speed up the training process. Set the sampling rate MR, get the compressed image dimension M = N × MR, and then we can generate a random Gaussian matrix of [M, N] and orthogonalize its vector to get the measurement matrix. Use the measurement matrix to sample *x* for compressed sensing to obtain the corresponding measurement value y.

### 3.2. Initial Reconstructed Image

**Figure 4:**
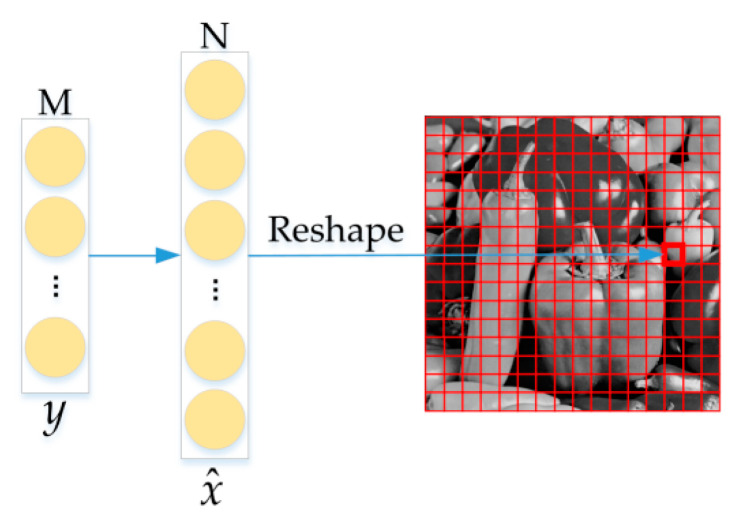
The process diagram of initial reconstructed image.

Since the mapping from the measurement vector y to the original signal x can be regarded as an approximately linear mapping, *x* = Ay, where A ∈ *R*^M×N^ is a linear mapping matrix, although the equation belongs to the overdetermined equation without an exact solution, we can estimate a mapping matrix A’ to approach A infinitely. Therefore, input the compressed image to the linear mapping network to obtain the initial reconstructed image $\hat{x}$. The essence of the linear mapping function is a fully connected layer; its mathematical model is shown in Equation (4). The initial reconstruction process is shown in Figure 4. To ensure that the initial reconstructed image is consistent with the original crop image, set the number of neurons in the fully connected layer to N.

(4) $$H_{u,b}(u)=f(w^{T}u+b)$$

where *u*, *w*, *b*, *H_ub_*(*u*), *f*() represent the feature vector of image information, weight vector, biases, output of fully connected layer, and activation function, respectively.

### 3.3. Multi-Feature Residual Network

The agricultural environment is usually more complex, and the acquired images often have complex backgrounds such as uneven lighting. The extraction and analysis of the texture features of the images can be used as an important basis for evaluating the growth status of crops. Therefore, in this paper, convolution kernels of different scales are used to convolve the image separately to capture more different details of image feature information, so that the image information is more completely retained, and the image reconstruction ability of the network is improved.

**Figure 5:**
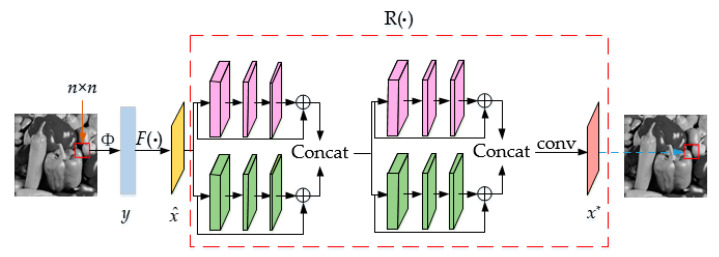
Schematic diagram of the MRNet.

The linear mapping network is recorded as the function F(·). According to the analysis described above, with input the original image *x* to the function F(·), we can obtain an initial reconstructed image $\hat{x}$ in order to reduce the error between the two and achieve a better reconstruction effect; multi-feature depth residual reconstruction is performed on $\hat{x}$, and it is recorded as a function R(·). Specific method: first, input $\hat{x}$ into two residual modules of different scales; second, tensor stitching is performed on the output of the two modules, which is recorded as $x_{1}$; then, send $x_{1}$ to residual modules of different scales and perform tensor stitching; finally, 1 × 1 convolution is performed to obtain the final reconstructed image x*. The specific mathematical model is shown in Equation (5). The compressed sensing image reconstruction model based on the multi-feature residual network is shown in Figure 5.

(5) $$x^{*}=F(y,W^{1})+R[F(y,W^{1}),W^{2}]$$

where *W*^1^ is the weight matrix in the linear mapping network and *W*^2^ represents the weight matrix in the multi-feature residual network.

In the multi-feature residual network, in order to maintain the dimension of the image feature information, the network does not contain a pooling layer, only the convolution layer and the activation function. The “same” setting is performed during convolution. In this paper, we choose the rectified linear unit (ReLU) as our activation function. The value of ReLU in the negative interval is 0, and the value in the non-negative interval is constant. Therefore, there is no problem of gradient disappearance when the network parameters are back propagated, so it is convenient to use the back propagation algorithm in deep artificial neural network training. At the same time, it can ensure that the convergence speed of the network model is maintained in a relatively stable state.

## 4. Experiment and Analysis of Results

In our paper, the experiment is implemented in Intel(R) Core(TM) i5-6200U CPU @2.30GHz of Lenovo laptop which was made in Beijing, China, Windows 10 operating system, and the simulation software is PyCharm which is developed by JetBrains in Prague, Czech Republic. In order to verify the effectiveness of the proposed multi-feature residual network model in solving crop image reconstruction problems, the same settings were used in the deep learning model training to compare the reconstruction effects of the test images.

### 4.1. Experiment and Parameter Setting

This paper chooses mean squared error (MSE) as the loss function of learning and training. Its mathematical model is shown in Equation (6):

(6) $$MSE=\frac{1}{N}\sum_{i=1}^{N}[y^{i}-f(x^{i}){]}^{2},$$

where *N* represents the total number of samples, *y^i^* represents the *i*-th true value, and *f(x^i^)* represents the *i*-th estimated value.

MSE is the expected value of the square of the difference between the estimated value of the image and the true value of the image. It can evaluate the similarity between the reconstructed image and the original image. In a certain sense, the smaller the value of MSE, the higher the quality of the reconstructed image. Therefore, the Adam optimizer is used to update and calculate the network parameters to minimize the loss function.

For image reconstruction algorithms, the quality and similarity of the reconstructed image is a key evaluation factor for the pros and cons of the network model. We select peak signal to noise ratio (PSNR) and SSIM to quantitatively analyze the quality of the reconstructed image. A larger PSNR indicates a smaller image distortion. SSIM measures the similarity between the reconstructed image and the original image. If SSIM equals 1, the two images are identical.

**Figure 6:**
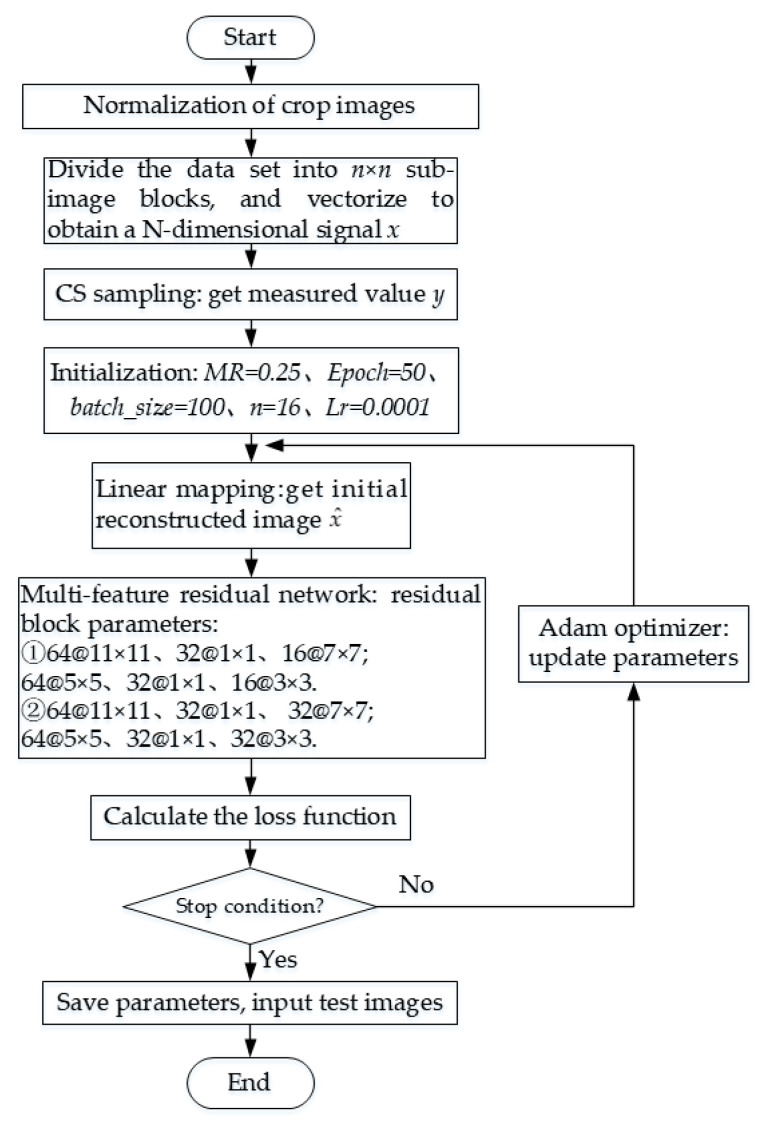
The flow chart of the experiment.

In summary, the specific implementation steps and parameter settings of the method are shown in Figure 6:

### 4.2. Results

In view of the characteristics of crop images, such as complex image environment, the model proposed in this paper uses multi-scale convolution to extract more feature information. In practical applications, crop images obtained by WSNs should be selected for targeted training to make it more suitable for a single field. In order to verify the universality and effectiveness of the model, this paper selects the universal dataset Set91 [19], which contains a variety of image information. After data enhancement, a total of 32,000 16 × 16 subimage blocks are selected. First, test on the benchmark data set Set11 [19], and then select three crop images for testing: cotton (256 × 256), little_pepper (256 × 256) and tomatoes (256 × 256). The comparison experiments are DR2-Net, CSRNet, and MSRNet in the past three years. In order to ensure the validity of the comparison results, all experiments use the same dataset, sampling rate, measurement matrix, learning rate, and epoch for training and testing. Note that the epoch was set at 50 to shorten the training time.

The parameter selection in this paper has a certain universality. The four images in the experiment use the same set of parameters, and this set of parameters can also be extended to the reconstruction of other crop images. Table 1 shows the PSNR and SSIM of each image reconstructed by various algorithms at a sampling rate of 0.25. SSIM measures the image similarity in terms of brightness, contrast, and structure. Both the proposed algorithm and MSRNet use multi-scale convolution to obtain image features. The reconstruction effect of the two is superior to other single feature reconstruction algorithms, indicating the effectiveness for the extraction of multi-feature information.

**Figure 7:**
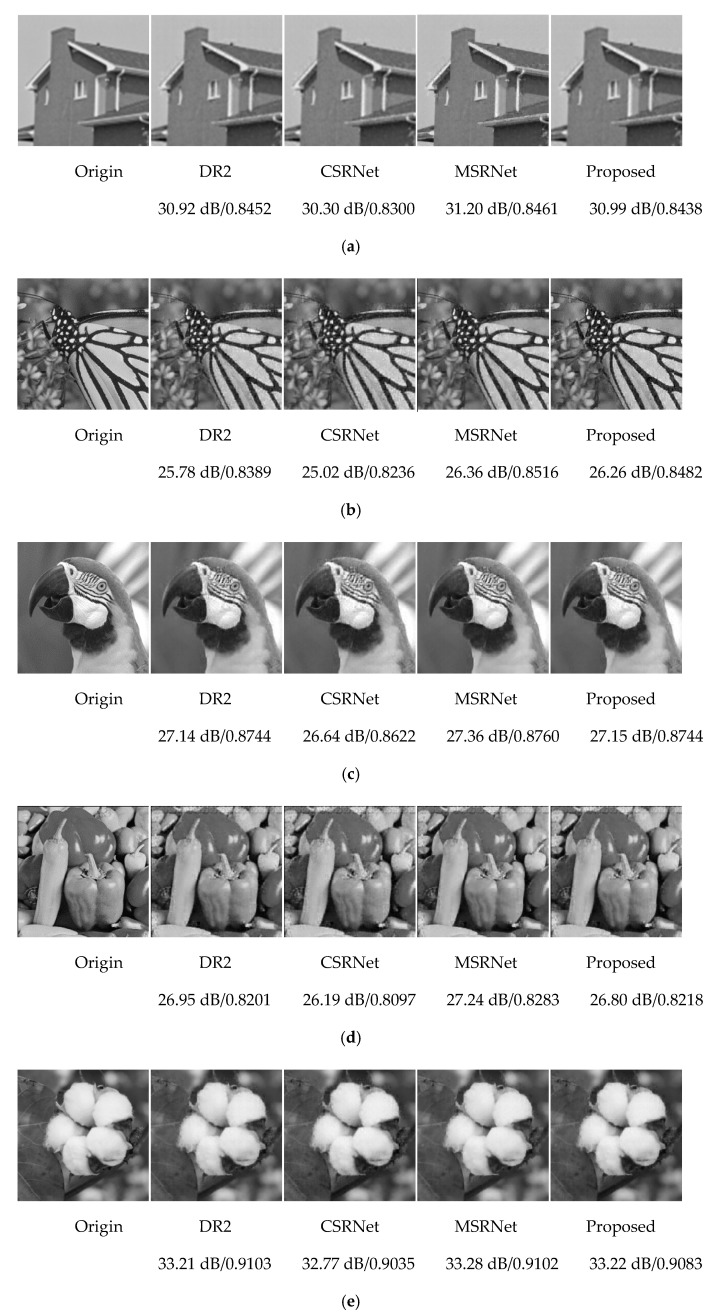

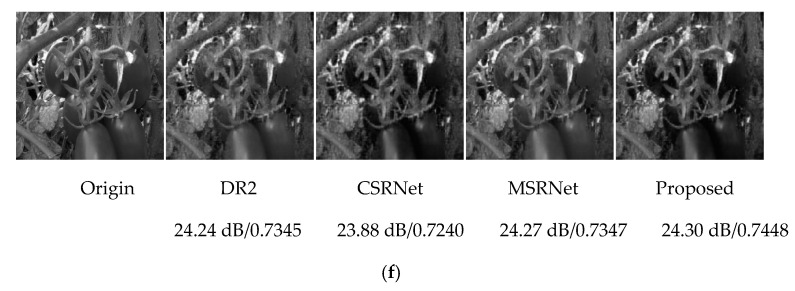
Several reconstructed images using different algorithms: (**a**) house, (**b**) monarch, (**c**) parrots, (**d**) pepper, (**e**) cotton, and (**f**) tomatoes.

Through a comparison between PSNR and SSIM, we find that the large PSNR value of the image does not mean that the SSIM is necessarily high, and its value can only represent the image quality to a certain extent. There are two main goals in this study of image reconstruction: one is to analyze using images whether the crops are growing in good condition and the other is to achieve the visual presentation of the crop growth process. Therefore, objective and subjective evaluation criteria are required to jointly evaluate the image quality. Figure 7 shows the reconstruction results of some representative test images.

Table 2 shows the average time of all images reconstructed by different algorithms at MR = 0.25. It represents the reconstruction efficiency of the algorithm.

**Figure 8:**
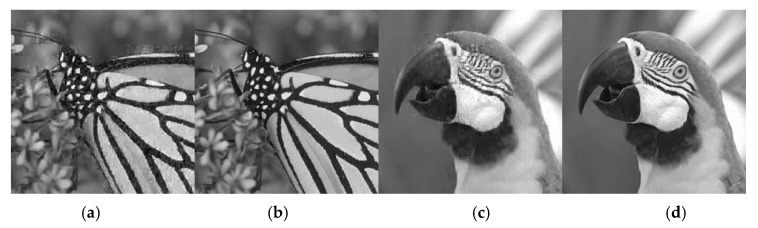
Reconstructed images of monarch and parrots in the size of 256 × 256 and 512 × 512: (**a**) 26.26 dB/0.8482, (**b**) 33.06 dB/0.9272, (**c**) 27.15 dB/0.8744, and (**d**) 34.20 dB/0.9525.

During the experiment, it was found that inputting images of different sizes into the network can acquire different PSNR and human visual perception. As shown in Figure 8, the larger images can get a better reconstruction effect. In testing, the size of images is 256 × 256, the average PSNR, SSIM, and time of the reconstructed image are 27.48 dB, 0.8370 and 2.4 s, so we completed relatively high-quality image reconstruction faster. The texture is relatively clear, generally, the extraction of texture and crop color can be used as an important basis for judging their growth status. Therefore, the algorithm of this paper reached the target requirements. The future work is to reconstruct color images, which can provide complete image analysis and display.

## 5. Conclusions

This paper proposes a deep compressed sensing image reconstruction method based on a multi-feature residual network. By continuously adaptively adjusting the network parameters, and adaptively learning the structural characteristics of the image, the assumptions of the original signal sparseness of the CS theory are relaxed; converting the traditional measurement and reconstruction process into an end-to-end mapping network greatly reduces the image reconstruction operation time, that is, using a small amount of data to accurately reconstruct crop image information in a short time. Experimental results show that the method proposed in this paper offers a good reconstruction effect on the test image and can obtain high-quality reconstructed images. The future research work is to realize the reconstruction of crop images in the color space, perform image processing analysis while monitoring the growth situation of crops, and predict the development status of crops.

## 6. Patents

Guiling Sun, Ruili Nan, Zhihong Wang, Shijie Wang. Research on Image Reconstruction of Compressed Sensing Based on Multi-feature Residual Network. China. 2020105099841, 8 June 2020.

## Figures and Tables

**Table 1:** The peak signal to noise ratio (PSNR) (dB) and structural similarity (SSIM) in the range of [0,1] on the reconstructed images.

Images	DR2	CSRNet	MSRNet	Proposed
barbara	25.27/0.7992	24.62/0.7679	25.32/0.7930	25.25/0.7953
boats	28.93/0.8413	28.41/0.8291	29.16/0.8477	28.91/0.8422
cameraman	24.71/0.7874	24.24/0.7775	24.89/0.7932	24.68/0.7894
fingerprint	25.85/0.9045	25.54/0.8983	25.74/0.8998	25.86/0.9046
flintstones	23.61/0.7482	22.95/0.7297	24.08/0.7609	23.45/0.7455
foreman	32.31/0.8902	31.78/0.8830	32.70/0.8932	32.49/0.8896
house	30.92/0.8452	30.30/0.8300	31.20/0.8461	30.99/0.8438
lena	28.04/0.8506	27.47/0.8357	28.29/0.8505	28.09/0.8508
monarch	25.78/0.8389	25.02/0.8236	26.36/0.8516	26.26/0.8482
parrots	27.14/0.8744	26.64/0.8622	27.36/0.8760	27.15/0.8744
pepper	26.95/0.8201	26.19/0.8097	27.24/0.8283	26.80/0.8218
cotton	33.21/0.9103	32.77/0.9035	33.28/0.9102	33.22/0.9083
little_pepper	27.02/0.8597	26.77/0.8479	27.12/0.8586	27.21/0.8586
tomatoes	24.24/0.7345	23.88/0.7240	24.27/0.7347	24.30/0.7448
Average	27.43/0.8360	26.90/0.7641	27.64/0.8388	27.48/0.8370

**Table 2:** Time consumption of reconstruction process.

Algorithm	DR2	CSRNet	MSRNet	Proposed
Average time	3.4137	1.4145	3.3250	2.4066
